# Next-generation sequencing: unraveling genetic mechanisms that shape cancer immunotherapy efficacy

**DOI:** 10.1172/JCI154945

**Published:** 2022-06-15

**Authors:** Ahmed Halima, Winston Vuong, Timothy A. Chan

**Affiliations:** 1 Department of Radiation Oncology, Taussig Cancer Institute, and; 2 Center for Immunotherapy and Precision Immuno-Oncology, Cleveland Clinic, Cleveland, Ohio, USA.; 3 National Center for Regenerative Medicine, Cleveland, Ohio, USA.

## Abstract

Immunity is governed by fundamental genetic processes. These processes shape the nature of immune cells and set the rules that dictate the myriad complex cellular interactions that power immune systems. Everything from the generation of T cell receptors and antibodies, control of epitope presentation, and recognition of pathogens by the immunoediting of cancer cells is, in large part, made possible by core genetic mechanisms and the cellular machinery that they encode. In the last decade, next-generation sequencing has been used to dissect the complexities of cancer immunity with potent effect. Sequencing of exomes and genomes has begun to reveal how the immune system recognizes “foreign” entities and distinguishes self from non-self, especially in the setting of cancer. High-throughput analyses of transcriptomes have revealed deep insights into how the tumor microenvironment affects immunotherapy efficacy. In this Review, we discuss how high-throughput sequencing has added to our understanding of how immune systems interact with cancer cells and how cancer immunotherapies work.

## Introduction

The role of genetics in determining cancer immunotherapy response is born from two fundamental principles: (a) cancer is at its core a genetic disease, and (b) genetic mechanisms underlie the ability of the immune system to recognize diverse targets. These concepts evolved alongside the growing capabilities of next-generation sequencing (NGS) technologies that were increasingly used to identify targetable oncogenic driver mutations with the intent to expand the utility of genetic profiling in the clinic. The enthusiasm for this approach in immuno-oncology was amplified by the observation that the expression of the immune checkpoint inhibitor targets alone — i.e., PD-1, PD-L1, and CTLA-4 — was really quite insufficient to optimally identify patients with cancer who do or do not respond to immune checkpoint blockade (ICB) (1, 2).

The hypothesis that tumor genetics can influence immunotherapy response in patients with cancer was first observed in the setting of anti–CTLA-4 therapy for metastatic melanoma (2). It was observed that tumor mutational burden (TMB), which reflects the number of immunogenic neoantigens, was predictive of therapeutic response. Following the demonstration of this foundational principle — that alterations of the somatic genetic landscape can directly affect immunogenicity — biomarker development in immuno-oncology expanded rapidly. Investigations examining the effect of many types of genetic alterations — individual gene mutations, mutational signatures, genomic instability, and host genetics — have revealed a constellation of factors that influence the efficacy of ICB (3).

Clinically, immune checkpoint inhibitors (ICIs) have improved outcomes in oncology, including prolonging the survival of patients across various cancers whether alone or in combination with other agents (4). Despite the progress made, the majority of patients do not benefit from ICIs. Our ability to predict therapeutic benefit has improved via development of tumor- and patient-specific biomarkers in recent years; however, much improvement is needed. For instance, PD-L1 IHC is the most widely used biomarker in clinical practice for patients receiving ICB; however, many tumors with high PD-L1 expression do not respond to PD-1/PD-L1 inhibitors. Furthermore, there is an unmet clinical need to develop combinatorial biomarkers that integrate multiple determinants of response to more effectively predict ICI response above and beyond what is achievable with single biomarkers. In this Review, we acknowledge the vast and diverse efforts toward satisfying this unmet need and highlight biomarker development through the lens of genomics, given its integral role in identifying clinically relevant biomarkers as well as its potential for furthering our understanding of immuno-oncology.

## Tumor cell–intrinsic determinants of immunotherapy response

### TMB.

TMB most commonly refers to the number of nonsynonymous single-nucleotide variants (nsSNVs) in a tumor. It can be assessed by multiple NGS techniques, including whole-exome sequencing (WES), whole-genome sequencing, or NGS panels that sequence predetermined sets of cancer-related genes, such as MSK-Impact, Tempus xT, and the FoundationOne CDx panel. Our group first discovered that the presence of high TMB or mutations in DNA damage repair genes is likely to respond better to ICI treatment (2, 5–8). High TMB (>100 nonsynonymous mutations per exome) and the presence of specific neoepitope signatures correlated with response to and survival with CTLA-4 inhibitors in a discovery set of 25 patients and a validation set of 39 patients with melanoma. Patients with a high versus low TMB had a 50% versus 10% survival at 40 months in the discovery set (9). In June 2020, the US FDA approved pembrolizumab (anti–PD-1) for treatment of patients with malignant solid tumors of any type given 10 or more mutations per megabase (TMB ≥10), based on the KEYNOTE 158 study (10). In this prospective trial, 102 of 790 evaluable patients had tumors with a TMB ≥10. Patients with tumors with TMB ≥10 demonstrated significantly greater response rates (29% vs. 6%) than patients with tumors with TMB less than 10. In an analysis of 1678 anti–PD-1/PD-L1–treated patients with 16 different solid tumors, 25% of patients were found to have a high TMB using the TMB ≥10 cutoff. TMB-high tumors had higher response rates than TMB-low tumors in 11 of the 16 cancers. Using a cancer type–specific cutoff, TMB-high tumors showed numerically higher response rates in 14 of 16 cancers (11). Associations between high TMB and ICI response have been reported across various cancers (12), including urothelial carcinoma (13), small cell lung cancer (14), melanoma (9), and non–small cell lung cancer (NSCLC) (5). Numerous studies have validated our initial findings and, together, unequivocally validated that TMB helps drive ICI benefit.

As larger cohorts of patients are treated with ICIs, it is becoming evident that the prognostic utility of TMB is context dependent; ICI-treated patients have a better outcome with high TMB, whereas non-ICI-treated patients could potentially have worse prognosis with high TMB (8). Interestingly, frameshift insertions/deletions (fs-indels), although less common, are thought to be highly immunogenic mutations (15). These are usually degraded through the nonsense-mediated decay pathway. However, a fraction of fs-indels can escape degradation and have been found to predict response to ICIs even in TMB-low tumors (16). While TMB has provided the ability to select for patients likelier to respond to ICIs, better prediction approaches will necessitate the use of this biomarker in conjunction with others. Furthermore, harmonization of TMB reporting is needed, and substantial efforts are under way to accomplish this (17).

### PD-L1 expression.

IHC measurement of tumoral PD-L1 is one of the most commonly used clinical tests in guiding the use of anti–PD-1 and anti–PD-L1 therapy. Several conflicting studies reported both positive correlation and no correlation between PD-L1 expression and ICI response, potentially owing to variable PD-L1 staining (14, 18–25). A meta-analysis by Liu et al. of 24 randomized trials of patients who received PD-1/PD-L1 blockade found an improved overall survival (OS) in both PD-L1–positive and –negative patients. The magnitude of benefit was dependent on the expression of PD-L1 (26). In addition to its role as an immunosuppressive molecule, PD-L1 has several tumor-intrinsic roles in cancer initiation, metabolism, inhibition of proapoptotic signals, tumor growth, epithelial-mesenchymal transition, and metastasis through downstream signaling (27–29). Although the focus of this Review is not on PD-L1 IHC, it is nonetheless important to mention that PD-L1 staining and high TMB are not identifying identical sets of patients. These biomarkers are complementary in their predictive value and can be used together to improve identification of ICI responders (30).

### Tumor neoantigens and immunogenicity.

Tumor neoantigens are somatic mutation–generated peptides presented on the surface of tumor cells and are absent from normal tissues (31). These neoantigens can be presented by major histocompatibility complexes (MHCs) and trigger a neoantigen-specific T cell receptor–mediated (TCR-mediated) response (32). Neoantigens are thought to arise as a result of genetic alterations such as nonsynonymous single nucleotide variants (nsSNVs), insertions/deletions (indels), recombination events, gene fusions, or defective mRNA alternative splicing (AS) (31). Defective AS can lead to neoantigens that might change temporally as the tumor splicing machinery evolves (33). WES can be used to identify candidate neoantigens produced from nsSNVs, gene fusions, or indels. However, WES may not capture subtler neoantigenic changes from posttranscriptional or splicing-related alterations (34). Combining NGS, RNA sequencing, and proteomics can improve neoantigen prediction, and several groups have elucidated features of mutated peptides that are likelier to be immunogenic (35, 36). Although neoantigen levels generally track with TMB, it is our opinion that the current generation of prediction algorithms does not have sufficient accuracy for routine use as biomarkers (37).

Neoantigenic immunogenicity requires MHC-neoepitope complex presentation and subsequent T cell recognition. Currently, a considerable challenge stems from our inability to distinguish the neoantigens that will trigger a significant T cell immune response from those that bind to MHC without a T cell response. Multiple issues contribute to this challenge. First, immunogenic neoantigens might only be expressed by a minority of tumor cells. Single-cell sequencing techniques might be able to dissect heterogeneous tumor neoantigen expression more precisely (38). This is important because both the degree of clonal frequency and the quality of a neoantigen seem to affect its immunogenic potential (39, 40). Second, surface density of MHC-neoepitope complexes appears to contribute to the extent of T cell activation, and this density is difficult to routinely measure (41). Third, even if these neoantigens are presented, TCR recognition is variable (3). One effort to formalize a model to determine neoantigen immunogenicity has been attempted by the Tumor Neoantigen Selection Alliance (TESLA), which has identified five features that can help differentiate immune recognition of MHC-I–restricted peptides generated by SNVs and indels (36): MHC binding affinity; binding stability; clonality; the differential between the MHC binding affinity of a mutated peptide and that of the wild-type form (i.e., agretopicity); and foreignness. This model does not take some critical factors into consideration, and its construction did not use modern learning approaches, so it is unclear how generalizable it is (42, 43).

In contrast, contemporary approaches to prediction of MHC-binding potentials are increasingly relying on state-of-the-art artificial intelligence algorithms, such as neural networks (NetMHC and ConvMHC), random forest classifiers (ForestMHC), and natural language processing techniques (HLA-CNN) (44–46). MHC binding predictors have markedly improved in the last several years. While these approaches may aid in more precise identification of MHC binding predictors, there remain many challenges to bridging this to routine clinical use, as highlighted by Pearlman et al. (47).

### Mutational signatures.

Tumors with mutagen-specific mutational signatures have been shown to respond to ICIs more favorably. For example, in patients with NSCLC treated with pembrolizumab, the smoking-related signature was associated with significantly improved progression-free survival (PFS) (5). Similarly, UV light–generated alterations that characterize melanoma are associated with favorable response to ICIs. In fact, the UV mutational signature has been shown to increase the hydrophobicity of neoantigens, making the neoantigens better presented by the MHC and better recognized by T cells (48). Distinct hypermutable states that result from mutations in mismatch repair (MMR) and DNA polymerase epsilon/delta (POLE/POLD1) genes are associated with improved response to ICIs (49). *POLE*/*POLD1* mutations result in hypermutability due to their role in DNA proofreading and fidelity during replication (50). MMR deficiency and POLE/POLD1 mutations lead to a high TMB, thus conveying a better response to ICIs (51). Because TMB and DNA repair deficiencies such as microsatellite instability (MSI) are causally linked to hypermutation signatures, the quality and quantity of mutation generation and subsequent neoantigen creation cannot be functionally separated. MMR deficiency has been approved by the FDA as a tumor type–agnostic biomarker for pembrolizumab and nivolumab. Furthermore, there are now prospective clinical trials of anti–PD-1 therapy in patients harboring mutations in *POLE*/*POLD1* that are not MSI-high (ClinicalTrials.gov NCT03810339). *APOBEC* mutational signatures have also been reported to be predictors of ICI response in urothelial cancer, NSCLC, and head and neck cancer (52–53). *APOBEC* is a family of cytidine deaminases that induce mutations in viral genomes, hindering viral replication. However, off-target activity on the host genome can also lead to a hypermutated status that has been shown in an in silico model to lead to increased hydrophobicity of the neoantigens, leading to more robust immune response (55).

### Microsatellite stability.

Mutations in the MMR pathway lead to MSI and a high number of somatic mutations, which generate a high number of tumor neoantigens (56). We first observed that ICIs can result in strong response in tumors with mutations in the MMR pathway (5). This observation was subsequently verified by a clinical trial of anti–PD-1 therapy that enrolled 41 patients with MMR-deficient colorectal cancer (CRC) (cohort A), MMR-proficient CRC (cohort B), and MMR-deficient non-CRC (cohort C). Response rates were 40%, 0%, and 71%, respectively (49). A later study of PD-1 blockade in 12 different MMR-deficient cancers showed 21% complete responses and 33% partial responses. These findings led to the tissue-agnostic FDA approval of anti–PD-1 immunotherapy for solid malignancies with MSI (57). A phase III randomized trial then led to approval of pembrolizumab as first-line therapy for advanced MSI^+^ CRC (58). Interestingly, 45% to 70% of MSI^+^ patients do not respond to ICIs (59). Differences in the degree of MSI present in tumors and in indel load (59, 60), disruptions in *WNT* signaling, and defective antigen presentation as a result of loss of β_2_-microglobulin or loss of HLA molecules are all likely contributors to differential responses in MSI^+^ tumors (61).

### Aneuploidy.

Aneuploidy or somatic copy number alterations (SCNAs) have been shown to be associated with worse outcomes after ICI treatment (62). More recently, specifically arm- and chromosome-level SCNAs were correlated with immune evasion in 10 of 12 cancers studied based on analysis of The Cancer Genome Atlas. This study also showed that tumor aneuploidy negatively correlated with survival in ICI-treated metastatic patients with melanoma in two clinical trials (63). Interestingly, total SCNA burden was not predictive of response. However, specific losses and amplifications were found to be predictive. For instance, 9p21 amplification was associated with resistance (64), while 9q34 loss was associated with higher response rates (65). Furthermore, SCNAs in *IFNG*, the gene encoding IFN-γ, were also found to be enriched in ICI-resistant patients with melanoma (66). Using an unbiased machine-learning method, Chowell et al. validated the effect of extent of copy number alteration on ICI response. The degree of effect is observable but smaller than that of TMB and some other factors (67).

## Mutations in discrete genes influencing immunotherapy outcomes

### BRAF mutations.

Mutant *BRAF* suppresses intratumoral T cells via overexpression of IL-1α and IL-1β by tumor cells, leading to overexpression of PD-L1 and PD-L2 in tumor-associated fibroblasts (68). Patients with melanoma with *BRAF* mutations had higher rates of PFS and OS when treated with combination PD-1 and CTLA-4 ICIs compared with patients with wild-type *BRAF* in the CheckMate 067 trial (68% vs. 53% 3-year OS, respectively; ref. 69). However, it is likely that other factors can modulate the overall influence of *BRAF* mutation. In a study of 68 patients with melanoma who received nivolumab, pretherapy TMB and clonal mutation load correlated with survival and response in patients who were ipilimumab naive, but not in those who previously progressed on ipilimumab. Context is therefore an important factor influencing the effects of these biomarkers.

In patients with melanoma with *BRAF* V600 mutation, ICIs and tyrosine kinase inhibitor therapy are both potential options. Which is the better therapy to use in the first-line setting? The DREAMseq trial recently showed that patients with *BRAF* V600–mutated advanced melanoma who received nivolumab plus ipilimumab followed by dabrafenib plus trametinib experienced greater OS (72%) compared with patients receiving the converse sequence (52%) (70).

### Mutations in KRAS and STK11/LKB1.

*KRAS* is the most frequently mutated oncogene in lung adenocarcinomas. *KRAS*-mutated lung adenocarcinomas can be subdivided into *STK11*/*LKB1*-comutated or *TP53*-comutated subtypes (71). In the Stand Up To Cancer (SU2C) cohort of 174 patients treated with nivolumab, patients treated with nivolumab with *STK11*/*LKB1* comutation had significantly lower objective response rates (7.4% vs. 28.6%), median PFS (1.8 vs. 2.7 months), and median OS (6.4 vs. 16.1 months) compared with the *KRAS*-mutated, *LKB1* wild-type patients. *STK11*/*LKB1* alterations were also significantly associated with PD-L1–negative status in lung adenocarcinomas with intermediate to high TMB. In addition, patients with positive PD-L1 also had worse outcomes if they had an *STK11*/*LKB1* alteration (71). The interaction of *KRAS* and *STK11* has also been demonstrated to have prognostic importance across cancers as coalteration of these genes is associated with overall worse prognosis (72). Therefore, these alterations may have both prognostic and predictive implications.

### PTEN.

*PTEN* loss of function (LOF) is suggested to decrease T cell infiltration via overexpression of immunosuppressive cytokines in a melanoma preclinical model (73). Analysis of 135 resected melanoma regional metastases found that melanomas with *PTEN* loss had significantly lower CD8^+^ T cell tumor infiltration than tumors with *PTEN* expression (74). *PTEN* loss causes overactivation of the *PI3K*/*AKT* pathway, which promotes immune evasion in various ways, such as sustaining the function of regulatory T cells (75).

### SWI/SNF complex, PBAF, and PBRM1.

Several genomic studies have identified various components of the *SWI*/*SNF* chromatin remodeling complex, such as polybromo- and BRG1-associated factors (*PBAF*), which contains *PBRM1*, *ARID2*, and *BRD7*, as frequently mutated genes across cancer types (76). *PBAF* complexes are ATP-dependent chromatin remodelers that regulate transcription (76).

*PBRM1* is the second most commonly mutated gene in clear cell renal cell carcinoma (ccRCC) after *VHL* and is a component of *SWI*/*SNF* chromatin remodeling complexes (77). In a cohort of 62 patients with ccRCC, those who had *PBRM1* LOF mutations responded better to ICIs (78). Preclinical data suggest that inactivation of components of the *PBAF* complex help overcome resistance to T cell–mediated cell killing in melanoma (79). However, several studies have shown conflicting implications of *PBRM1* mutations in ICI response across different cancers, i.e., no association or worse response through the conferring of a nonimmunogenic phenotype (80–83). These conflicting results are difficult to reconcile but might be explained by the presence of confounding factors in these studies or the weaker effects of *PRBM1* mutation on tumor immunity. Indeed, these studies were done before modern systematic efforts to quantify biomarker contribution to ICI response (67). Since it was done at an earlier time, the original study implicating *PBRM1* did not account for these factors (78).

### CDKN2A.

LOF mutations in *CDKN2A* were recently found to be associated with decreased response to ICIs in patients with NSCLC despite high PD-L1 expression and high TMB (84). Similarly, genomic alterations in *CDKN2A* were reported to be associated with a poor response to ICI in urothelial carcinoma in two large cohorts of patients. RNA sequencing data revealed decreased expression of genes involved in immune and inflammatory pathways in patients with *CDKN2A* alterations, which could provide an explanation for the deleterious effect of these mutations on response to ICIs (85).

### BRCA1 and BRCA2.

*BRCA1* and *BRCA2* mutations are responsible for defects in homologous recombination–based DNA repair (86). Interestingly, *BRCA1* and *BRCA2* were shown to cause distinct changes in the tumor immune microenvironment, with *BRCA2*-mutated tumors having higher gene expression of both adaptive immunity– and innate immunity–related pathways. These differential effects on the tumor microenvironment (TME) lead to contrasting outcomes of ICI treatment (6). *BRCA2* mutation was associated with better response and survival after ICI treatment in both patients and mice, whereas *BRCA1* mutation did not demonstrate these effects. Single-cell sequencing of tumors with these mutations showed differences in myeloid cells that likely regulate the TME. A recent clinical trial (CheckMate 650) treating prostate cancer with nivolumab and ipilimumab validated previous observations on the effects of homologous recombination repair deficiency on ICI response (6, 87). It is important to note that this trial also validated the effects of TMB on ICI response in prostate cancer.

### WNT/β-catenin signaling.

Activation of the WNT/β-catenin signaling pathway in melanoma has been correlated with T cell absence in human metastatic melanoma samples (88). The presence of pretreatment T lymphocytes and higher expression of a number of immune-related genes was shown to predict a better outcome to ipilimumab in patients with melanoma (89). Thus, it can be hypothesized that by decreasing T cell infiltration of melanomas, activation of β-catenin signaling can lead to decreased efficacy of ICIs. Large-scale sequencing of patients treated with ICIs also shows that tumors with WNT pathway mutations are associated with worse survival (6).

## Tumor cell–extrinsic determinants of response

Along with the ongoing examination of tumor-intrinsic biomarkers, there is a rapidly growing body of evidence supporting the important effects of tumor-extrinsic factors such as host germline variation, HLA genetic variation, the microbiome, and variation in the immune microenvironment. One related observation is the finding that 15% to 20% of the variation in intratumoral immune signaling via interferon and cytotoxic cell infiltration may be explainable by heritable variants in genes including *IFIH1*, *STING1*, *TMEM108*, and *RBL1* (90). At the interface of the immune cell–tumor cell interaction is an immune synapse dependent on the generation of an immunogenic antigen and its subsequent presentation via the HLA-I molecules. Just as genetic variations in the cancer genome affect the generation of these tumor peptides, variations in HLA genotypes and diversity shape how these peptides are used via immunopeptidome presentation. This ultimately shapes immunotherapy response (91). HLA-I molecules are encoded by *B2M* and the HLA-I genes (*HLA-A*, *-B*, and *-C*). The *HLA* gene family is the most polymorphic set of genes in the human genome (92). Polymorphisms of these genes are concentrated within the peptide-binding domains (93). Diversity in these peptide-binding domains leads to diversity in the presented peptides, which is selected for over evolution (94). HLA-I evolutionary divergence (HED) is a measure of diversity of physiochemical sequence divergence of the HLA molecules. In a cohort of mostly patients with melanoma who received ICIs, HED was a determinant of response and survival (91).

The power in measuring HLA-I variation seems to extend to correlation of benefit with some combination therapies, e.g., combination of tyrosine kinase inhibitors with ICIs in RCC (95). HED predicts aplastic anemia outcomes, bone marrow transplant outcomes, and organ transplant outcomes (96–98). In a meta-analysis of factors that influence ICI efficacy, Litchfield et al. also showed that HLA divergence was a significant factor influencing ICI response in melanoma (64). If HLAs are not actually typed but are estimated by imputation using microarrays, associations may be not apparent (99). Based on more recent modeling data, it appears that HED has a somewhat weaker effect compared with TMB, treatment beforehand with chemotherapy, or neutrophil/lymphocyte ratio. This may explain variation in predictive value between cohorts that are not balanced in all or some of these variables (67).

### Tumor immune microenvironment.

While host- and tumor-specific genetic factors form a basic blueprint for immunogenicity, the impact of the immune system is heavily dependent on the local microenvironment, the functional capacity of immune cells, and the balance between immunostimulatory and immunosuppressive stimuli (100–102). Efforts to expand our understanding of these interactions include the development of new computational and high-throughput approaches for immune cell profiling (103–107). These strategies include characterization of the dynamics of the microenvironment in which TCR repertoires may vary, immune-enriched and immune-desert regions that can exert regional immunomodulatory effects, and the influence of the microbiome (1, 3, 108–111). Studies have found that the density of tumor-infiltrating lymphocytes (TILs) can associate with OS regardless of whether patients are treated with immunotherapy (112). An early study found that lack of CD8^+^ T cells from the vicinity of tumors is associated with poor outcomes in CRC (113). More recently, this finding was refined into a parameter called the Immunoscore, in which T cells are quantified in different vicinities of tumors. This assay was found to be a strong predictor of OS in CRC (112, 114–116).

Tumors can be classified according to the degree of infiltration into various categories: immune-inflamed, immune-excluded, and immune-desert types. Immune-inflamed tumors are characterized by the presence of a dense infiltrate of favorably positioned CD4^+^ and CD8^+^ T cells in the proximity of tumor cells. Immune-excluded tumors have CD8^+^ T cells that are excluded from the tumor parenchyma and instead found in the peritumoral stroma. This phenotype is associated with increased TGF-β signaling in tumor fibroblasts (117). Finally, immune-desert tumors lack the presence of T cells in both the tumor parenchyma and stroma (118, 119). In addition, the exact localization of T cell infiltrate in tumors was found to be associated with response to ICIs. For example, the presence of a dense infiltrate of CD8^+^ T cells at the invasive margin as opposed to the center of the tumor was associated with response to ICIs (1). Forays into the contribution of different cellular compartments of the microenvironment via spatial transcriptomics have revealed increasing evidence for the role of B cells, unique CD8^+^ T cells, and tertiary lymphoid structures in the immunotherapy response across various diseases, including melanoma, sarcoma, and RCC (120–122).

Other characteristics, such as the intratumoral and peripheral immune cell receptor repertoire, which can be quantified with metrics such as entropy, richness, and clonality of T cell and B cell populations, were also associated with response to ICIs (123–127). The TCR repertoire was shown to be positively associated with polymorphism of HLA-I loci and negatively associated with CMV positivity and age (128). However, there are conflicting data on the effect of the TCR repertoire on the response to ICIs, likely owing to the complex interactions of the TCR repertoire with ICIs, patient immunologic histories, and environments (110, 129–131). Efforts to understand these conflicting findings have focused on identifying immune cell functional states via differentiation and lineage commitments as well as receptor specificity and clonality in both bulk and single-cell sequencing studies (132–135). Here, conflicting results do not indicate deficiencies in any particular study. Rather, we are likely observing associations affected by population variation and factors we don’t yet fully understand. Furthermore, the aforementioned factors at play in the TME are not yet routinely assessed in a clinical pathology setting and so remain hypothesis-generating given the current lack of standardized validation. It is perhaps most useful to develop combination biomarker suites or nomograms that take into account multiple major biomarkers.

### Development of combinatorial approaches to understand immunotherapy efficacy.

**Figure 1:**
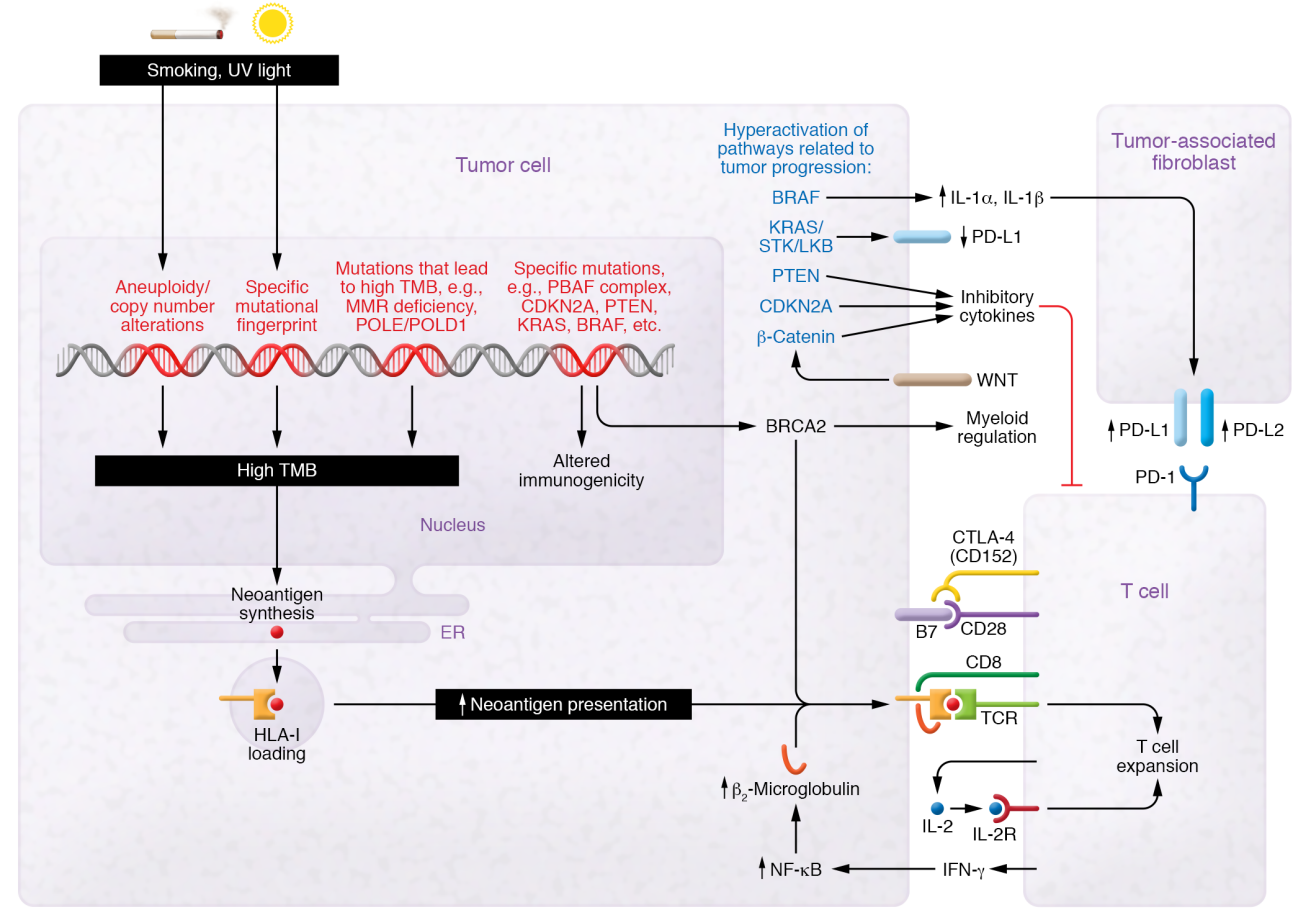
Genetic alterations and immunologic consequences. Certain mutagens, such as UV light and carcinogens in cigarette smoke, can lead to formation of mutations and aneuploidy, which can cause a high tumor mutational burden (TMB). Other specific alterations can lead to microsatellite instability or *POLE*/*POLD1* mutation, which results in hypermutation. This can result in transcription and translation of tumor neoantigens. These are presented on HLA-I molecules. HLA-I is required to present tumor neoantigens to cytotoxic T cells. Certain mutations, such as *KRAS*/*STK*/*LKB* in lung cancer, have been associated with decreased PD-L1 expression on tumor cells. Other mutations in genes such as *BRAF*, *CDKN2A*, and *PTEN* as well as aberrant activation of the WNT/β-catenin pathway have been implicated in increasing the release of inhibitory cytokines in the tumor microenvironment that act on tumor-infiltrating T lymphocytes or tumor-associated fibroblasts. T cells recognize antigens presented on HLA-I as “non-self” antigens; costimulatory signals are needed for Th cell activation. Costimulatory signals involve the binding of B7 on tumor or antigen-presenting cells to CD28 on T cells. CTLA-4 (CD152) competes with CD28 for the binding of B7, thus inhibiting the necessary costimulatory signal needed. Owing to space constraints, this diagram is not comprehensive.

The examples above demonstrate how systematic investigations into immunotherapy biomarkers are enabled by improvement in NGS technologies. With more data available, it became possible to form hypotheses about how multifaceted molecular processes interact with the immune system. However, as more evidence arises, we approach a “long tail” of candidate biomarkers wherein the likelihood of an individual biomarker having substantial predictive capability is diminishing. Not every factor found to influence a biological process is suitable as a biomarker. Biomarkers should (a) be feasible for use in clinical settings, (b) be able to be efficiently measured using cost-efficient means, and (c) provide useful information for decision making in medical practice. Multiple large studies have now clearly shown that, among genomic biomarkers, TMB and a few other factors provide the largest pan-cancer predictive value for ICI response (64, 67). Other genomic alterations, like *STK11/LKB1* mutation, can be important for specific cancers or can further modulate immunogenicity. Thus, combinatorial predictive biomarkers are clearly needed, and systems for harmonizing the various validated biomarkers are necessary to improve clinical utility. Figure 1 summarizes some important processes discussed in this Review.

The promise of combinatorial biomarkers has been highlighted by large-scale efforts from pan-cancer cohorts. For instance, the combination of high TMB and low pretreatment neutrophil/lymphocyte ratio is correlated with greater benefit from ICI (136). These studies demonstrate that select factors can predict response rates across cancer types, although there may be context-specific biomarkers for which the balance and predictive ability of genomic alterations, the TME, TMB, HLA-I diversity, and other markers may vary by tumor type (64, 137). Some have proposed using a combinatorial model based on the triple axis of tumor neoantigens/microenvironment/checkpoints to explain the variance in outcomes of PD-1/PDL-1–directed therapy (137). A meta-analysis of the CPI1000^+^ cohort demonstrated that at least 80% of the significant biomarkers by tumor type were also significant in the overall pan-cancer cohort and that there were differential predictive potentials of each biomarker by tumor type, such as TMB between melanoma and urothelial carcinoma and loss of 9q34 between RCC and the rest of the cohort (64).

Given the marked complexity of biomarker integration, classic methods of multivariable modeling may need to be supplemented by newer machine-learning methods. The development of artificial intelligence and machine learning in medicine has exploded in recent years, with improvements in modeling and feature selection enabling better prediction of treatment outcome (45). For instance, our recent work in combinatorial biomarker development has enabled superior pan-cancer prediction of immunotherapy response over TMB alone using an exhaustive approach with random forest classifier modeling (67). In the CPI1000^+^ cohort meta-analysis, a decision tree model was also generated, albeit with gradient boosting–based algorithms (64).

### Implications of single-cell profiling technologies for biomarker development.

The use of single-cell profiling technologies has enabled an unprecedented view into the complex dynamics between intratumoral cellular subpopulations (138–140). Recent examples of these studies have identified unique cell types associated with ICI response. These include (a) an abundance of CD8A^+^ tissue-resident T cells and interferon-stimulated gene–high (ISG^hi^) tumor-associated macrophages in RCC; (b) tissue-resident macrophages contributing to remodeling of the microenvironment during ICI treatment in lung cancer; (c) distinct neoantigen-specific TILs with specific transcriptional states; and others (103, 105, 106, 108, 138, 141). While these have provided new insights into the composition of the TME, the implications of these findings have yet to translate into clinical use (45, 140, 142). Various cellular atlases have been defined via modern single-cell sequencing, and while they are an enormous research resource, there still remain challenges in the ability to translate these efforts to clinical utility (143, 144). The combination of single-cell sequencing technologies with improved pathologic sampling, single-cell resolution multispectral imaging modalities, and microfluidics will first require new computational tools for interpretation at the biological level and subsequent systematic implementation into clinical trials for robust correlative biomarker identification (138, 142, 145–148).

## Dynamic biomarker profiling

While there has been an improved effort for sequential profiling throughout therapy in recent clinical trials, many older studies have been limited to analyses of pretreatment sequencing. We are seeing now that longitudinal sampling paired with molecular profiling can identify peritreatment biomarkers of treatment sensitivity with potential to identify the emergence of adaptive or acquired resistance as well (149–151). Serial profiling has demonstrated that immunoediting is operative in patients treated with ICIs (110). In a study of the pan-cancer INSPIRE cohort, upregulation of *PLA2G2D* was identified as a marker of resistance to ICIs alongside *B2M* loss of heterozygosity and copy-number abundance (152). Interestingly, a study showed that enrichment of CX3CR1^+^CD8^+^ T cells early during ICI correlates with survival in patients with lung cancer and can be monitored peripherally via blood sampling (135). Longitudinal sampling can also unveil evolutionary dynamics of resistant cells with differential site-specific microenvironments, such as those of *NGFR*^hi^ versus *NGFR*^lo^ melanoma cells with differential effect based on PD-L1 expression levels (153). These are only a few recent examples of a vast body of studies that highlight different dynamic elements that change over the course of therapy and are ever-increasingly understood via improvements in single-cell and spatial profiling technologies (138, 142).

The sequencing of circulating tumor DNA (ctDNA) has shown promise for the ability to monitor response to ICI after initiation of treatment. Still, there remain substantial challenges to clinical implementation of dynamic circulating biomarker profiling, including lack of harmonization of reporting metrics, varying kinetics of response from study to study, and varying levels of sensitivity between different assay technologies (154, 155). Improvements in the near future may help make ctDNA sequencing useful for monitoring patients who have already begun treatment and may aid in treatment intensification or even deintensification. Pretreatment ctDNA analysis may also be useful for identifying mutations that can affect ICI sensitivity or calculate blood TMB, which has shown promise in predicting ICI sensitivity (156–158).

## Conclusion and future directions

The current state of biomarker development for cancer immunotherapy is excellent, with an ongoing data deluge of considerable potential and ever-improving technologies. Tumor-agnostic FDA approvals for immunotherapy have been achieved, which is a notable achievement in oncology. However, there is still work to do to improve predictive strategies for identifying responders and nonresponders to ICIs, especially in the setting of combination therapies. As discussed above, we are on a path toward understanding the interplay of relevant mechanisms intrinsic to the tumor, the host, and the interaction between tumor and host and how different processes may vary in a context-specific manner. Biomarker models need to be feasible for clinical use and require technological improvements that may be assisted by advances in artificial intelligence and machine learning. Our field’s understanding of immuno-oncology and immunotherapy response has come a long way very quickly, and there is no sign that it will be slowing down anytime soon.

## Version 1 06/15/2022

Electronic publication
